# Study on the Evolution of the Game of Willingness to Cooperate between Residents and Separation Enterprises in Waste Separation Considering the Convenience of Separation Facilities

**DOI:** 10.3390/ijerph20021149

**Published:** 2023-01-09

**Authors:** Lichi Zhang, Yanyan Jiang, Junmin Wu

**Affiliations:** 1 School of Economics and Management, Jiangsu University of Science and Technology, Zhenjiang 212000, China; 2 School of Electronics and Information, Zhenjiang College, Zhenjiang 212028, China

**Keywords:** separation facility convenience, distributivity, complexity, synergy, evolutionary game

## Abstract

The distributivity and complexity of separation facilities in waste separation cooperation are incorporated into the factors influencing the payoff of waste separation cooperation. The game payment matrix of waste separation cooperation is constructed based on the distributivity and complexity of separation facilities. The equilibrium solution of waste separation cooperation is obtained through the evolutionary game. The influence of different changes in distributivity and complexity of separation facilities on the willingness to cooperate in waste separation is explored through numerical analysis of cases. The study shows that when the distributivity of separation facilities is certain, the lower the complexity of separation facilities, the higher the willingness of residents and enterprises to cooperate; when the complexity of separation facilities is certain, the willingness of residents and enterprises to cooperate rises and then falls with the increase of distributivity of separation facilities; finally, when the distributivity and complexity of separation facilities change at the same time, the willingness of residents and enterprises to cooperate shows different changes with the different changes of two separation facilities convenience factors.

## 1. Introduction

Waste Separation (Garbage Classification) upgrades the traditional waste treatment method and is a scientific management method to dispose of municipal waste efficiently. With the increasing amount of waste and the urgent pressure of green development needs, it is increasingly necessary for residents and enterprises to cooperate in waste separation. The influencing factors on residents and enterprises cooperating in waste separation are complex. It is of practical significance to clarify the main influencing factors and mechanisms to promote waste separation and reduction, resource utilization, and overall improvement of resource efficiency.

At this stage, waste separation in China usually presents a situation where the government bears all the costs of waste separation. Meanwhile, the other stakeholders benefit from it without incurring costs, i.e., a “free-rider” problem [1] (Soltani et al., 2016). The free-rider problem will prevent the government from choosing more advanced and expensive technologies [2] or lead to overuse of the services provided. Waste separation requires the participation of several actors, of which residents and separation enterprises are essential participants [3,4] (Chen et al., 2017, Tang and You et al., 2017).

However, the effectiveness of waste separation depends on the source separation of residents [5] (Chen et al., 2018) and the post-processing of separation enterprises. Except for some first- and second-tier cities in China, most other regions are not sufficiently aware of residents’ participation in waste separation, so the efficiency of waste source separation is not high [6] (Wen et al., 2014). Separation enterprises relying on traditional treatment methods such as reduction and resourcefulness are low efficiency, and relying purely on residents [7,8] (Chen et al. 2020, 2022) or separation enterprises unilaterally, it is difficult to make a breakthrough in promoting waste separation. The separation enterprises redesign the waste separation business process to attract residents to separate waste at the source; residents actively participate in waste separation to reverse the transformation of the service mode of separation enterprises’ business, so the synergy between the two sides can effectively promote the improvement of waste separation effectiveness.

Zurbrügg and Drescher et al. [9] (2004) studied 17 decentralized waste separation collaborative systems in Indian cities such as Bangalore, Chennai, Pune, and Mumbai and summarized them into three categories: (1) citizen and community initiatives; (2) commercial and institutional initiatives operating in their locations; and (3) small and medium-sized private sector initiatives. Ordoñeza et al. [10] (2015) argued that Swedish housing companies can provide separation infrastructure designed for residents rather than just applying waste separation management systems. Agyeiwaah [11] (2019) conducted a qualitative study of structured interviews on collaborative waste separation from the perspectives of Japanese hotel accommodation-based businesses and occupants and found that sociocultural sustainability was the most relevant dimension, resulting in a hierarchy of sustainability relevance. Santti et al. [12] (2020) observed a collaborative pilot project in Finland. It was a waste separation collaborative activity established in August 2018 between the city of Kuopio, the student residence, the regional waste management and recycling company, and the Savonian University of Applied Sciences. Then the study found that through this waste separation, the collaborative activity increased the recycling rate of bio-waste from 76% to 97%, and the number of participating collaborative residents increased from 25% to 84%. Ling and Xu et al. ([13] 2017, [14] 2018, [15] 2021, [16] 2021) studied the active support and investment of real estate companies and community residents in Hangzhou, China, in increasing public participation and awareness of the importance of source waste separation played an essential role in increasing public participation and awareness of the importance of waste separation at source.

The factors influencing the cooperation between residents and separation enterprises in waste separation [17,18] (Chen et al., 2021; Jin et al., 2021) are complex, and it is essential to clarify the main influencing factors and their influencing mechanisms to improve the willingness of both parties to cooperate. This paper intends to analyze the mutual evolution law of cooperation between residents and enterprises in waste separation through evolutionary game theory and consider the influence of the convenience factor of separation facilities. This will explore how to formulate policies to promote the cooperation between residents and enterprises in order to improve the effectiveness of waste separation and provide theoretical reference for the harmless, reduction and resourcefulness of waste.

## 2. Literature Review

### 2.1. Research on the Influencing Factors of Waste Separation

Scholars have studied the factors influencing waste separation, Ekere et al. (2009) [19], Xu et al. (2016) [20], Stoeva and Alriksson (2017) [21], Shen et al. (2019) [22], Leeabai et al. (2019) [23], and Setiawan (2020) [24] stated that factors such as gender of participants, organizational membership, location, the topography of the location, suitable external conditions for waste separation, positive attitudes of participating subjects, clear definition of those responsible, years of education, practices, and perceptions have an impact on waste separation behavior. Razali et al. (2020) [25] found through their study that moral norms have an impact on waste separation, Lou et al. (2020) [26], on this basis, considered moral norms as a normative belief. The beliefs were divided into behavioral and normative beliefs and compared the impact on urban and rural residents’ waste separation under different beliefs. Wang et al. (2020) [27] found that perceived value and good separation facilities can increase residents’ satisfaction with waste separation, while Ma et al. (2020) [28] argued that policy tools have a more significant impact on waste separation than perceived value and considered policy tools a combination of separation facilities and information.

### 2.2. Research on Separation Facilities Factors

The factor of separation facilities is of essential influence in waste separation. The convenience of separation facilities was first considered to be an essential factor affecting household waste separation (Bernstad, 2014) [29]. Later, scholars have suggested that the lack of separation facilities (Setiawan, 2020) [24], ease of use, distance (Liu et al., 2020) [30], and being free of charge (Lou et al., 2020) [26] all have an impact on residents’ willingness to separate their waste. Using artificial intelligence technology on this condition, it shows a positive relationship between residents’ perception of environmental value, emotional value, and social value, and residents’ willingness to separate their waste. (Zhang, 2020) [31]. Leeabai et al. (2021) [32] found that the design of separation facilities has an impact on waste collection and the impact of separation behavior. They found that the three aspects of color, location, and ease of use of separation facilities had the most significant impact. Based on this, Jiang et al., (2021) [33] focused on the color and shape in the design of separation facilities and found it significantly correlated with the impact of waste separation when the color and shape of separation facilities matched.

Several of the studies mentioned above have focused on influencing factors and separation facility influences, and previously, the implementation of curbside waste separation programs was considered a key influence on whether households sorted their waste (Barr and Gilg, 2005 [34]; Timlett and Williams, 2008 [35]). In addition, there is a positive relationship between waste separation behavior and adequate space around the household to store recyclable separation facilities (Ando and Gosselin, 2005) [36], and insufficient space around the household to store recyclable waste is considered to be one of the factors contributing to the difference (Timlett and Williams, 2008) [35]. This may be an explanatory factor behind the findings of Timlett and Williams (2008) [35], which showed that households have higher participation rates in waste separation compared to public places, where storage space for waste separation is usually limited. However, the convenience and physical infrastructure of household waste separation facilities have been little investigated and discussed in the academic literature. To the authors’ knowledge, no studies have been conducted on the physical characteristics of waste separation facilities. Therefore, the purpose of this study is to focus on the convenience of separation facilities as a research direction and to consider a community of household collections as a waste separation subsystem, where the community generally consists of multiple households and the waste separation facilities installed in the community are usually considered to be storable and recyclable separation facilities around the household. In the microcosm of the waste separation subsystem, we further refine the convenience of the separation facilities around households in the community into distribution and complexity. The distribution refers to how many waste separation facilities are distributed in the community. The complexity refers to the number of people per 100 people in the community who find it challenging to use the waste separation facilities.

On the other hand, previous studies provide a favorable reference for the study of the convenience factor of separation facilities influencing waste separation cooperation, so this study introduces the convenience factor of separation facilities into the influencing factors of waste separation cooperation based on the related studies. Unlike previous studies, this study classifies the convenience factors of separation facilities into the distributivity and complexity of separation facilities and also considers the hardware basis of waste separation cooperation and the experience of cooperative subjects. In addition, this study investigates the influence of the convenience of separation facilities on waste separation cooperation by focusing on the willingness of waste separation cooperation.

The main reasons for using evolutionary games to study the willingness of waste separation for residents and enterprises to cooperate are the properties of public goods, the pollution generated by waste, and the non-competitive and non-exclusive nature of waste, as well as the “public tragedy” and “prisoner’s dilemma” arising from the process of waste separation (Nowak and Sigmund, 1993; Ott and Aoki, 2002) [37,38]. At the same time, the assumption of limited rationality in evolutionary game theory is more consistent with the behavior rules of both subjects of waste separation. In the process of cooperative waste separation between two parties, the single-subject game is a stochastic and shared repetitive learning game process, so the adjustment process of individual strategies can be modeled using replicated dynamic equations. Therefore, the evolutionary game analysis can reflect the subject’s evolutionary path and stable strategy.

This study constructs an evolutionary game payment matrix for residents and enterprises, and considers the effects of the two dimensions of separation facility distributivity and complexity on the benefits and costs of cooperation. This is done to find the evolutionary equilibrium of the cooperation game between the two parties and investigate the effects of different changes in the distributivity and complexity of separation facilities on the willingness to cooperate in waste separation through simulations and case studies.

## 3. Modeling

### 3.1. Model Constraints

In this study, the following constraints are based on the willingness to cooperate in waste separation by residents and enterprises, considering the factors influencing waste separation cooperation.

Constraint 1: Participating subjects. This study has two types of participants in waste separation cooperation: residents (A) and enterprises (B). Residents and enterprises pursue different interests and have different preferences in choosing cooperation strategies. Waste separation cooperation is also a dynamic game between residents and enterprises to reach optimal equilibrium.

Constraint 2: Cooperative strategy. The set of strategies for both residents and enterprises is {cooperation, non-cooperation}, and x and y represent the probability values of choosing the “cooperation” strategy at the initial stage of the evolutionary game for residents and enterprises, respectively. Conversely, (1 − x), (1 − y) represent the probability values of choosing the “non-cooperation” strategy at the initial stage of the evolutionary game for residents and enterprises, respectively x, y ∈ [0, 1].

Constraint 3: Distributivity of separation facilities. Separation facility distributivity is the degree of distributivity after implementing a cooperative waste separation project. The higher the distributivity of separation facilities, the easier it is for residents to get access to use separation facilities. In this study, the distributivity of separation facilities is expressed by the proportion of the total waste disposal points in the cooperative project. The distributivity of separation facilities in cooperative projects is expressed by λ.

Constraint 4: The complexity of separation facilities. The complexity of separation facilities is a vital indicator of the ease of operation of waste separation facilities. The higher the complexity of separation facilities, the more difficult and time-consuming it is for residents to use such separation facilities, the more time is needed, the higher enterprises’ promotion and publicity costs for such separation facilities in the early stage, and the higher the maintenance costs later. In this study, the complexity of separation facilities is expressed by the proportion of residents who think the operation of separation facilities is complicated compared to the total number of residents during the trial of separation facilities. The complexity of the separation facility before waste separation cooperation is expressed by ε. Then the separation facility convenience after the cooperation is where ε, λ ∈ [0, 1].

Constraint 5: Cooperative benefits. R denotes the theoretical benefit of residents and enterprises when they simultaneously choose the “cooperation” strategy. The cooperation benefit is influenced by the convenience of the cooperation separation facility, $\varepsilon^{\frac{1}{\lambda }}R$ denoted by the cooperation benefit. R > 0, β denotes the distributivity coefficient of residents in the cooperation benefit, the resident benefit is ${\beta \varepsilon }^{\frac{1}{\lambda }}R$, the enterprise cooperation benefit is $\left(1-\beta \right)\varepsilon^{\frac{1}{\lambda }}R$, β ∈ (0, 1).

Constraint 6: Cooperation cost. The convenience of separation facilities impacts the cooperative cost of waste separation for residents and enterprises. The higher the distributivity of separation facilities, the higher the cost of hardware invested by residents and enterprises, and the higher the cooperative cost. The higher the complexity of separation facilities, the higher the cost of use and maintenance by residents and enterprises, and the higher the cooperative cost. C denotes the total theoretical cost of residents and enterprises when they simultaneously choose the “cooperation” strategy. Using $\varepsilon^{\frac{1}{\lambda }}C$ to denote the cooperative cost invested by residents and enterprises, and α to denote the amortization coefficient of resident cooperative cost, the amortization cost of residents is ${\alpha \varepsilon }^{\frac{1}{\lambda }}C$. The amortization cost of enterprises is $\left(1-\alpha \right)\varepsilon^{\frac{1}{\lambda }}C$, α ∈ (0, 1).

Constraint 7: Penalty for breach of contract. A resident and a business enter into a cooperation agreement that sets forth the obligations of both parties to cooperate. This is denoted by P, the penalty for breach of the contract paid by one of the parties to the other.

Constraint 8: Government subsidies. The government supports the cooperation of residents and enterprises utilizing financial subsidies, denoted by S. The government’s financial subsidies for waste separation cooperation are denoted by S.

The payment matrix of waste separation cooperation of residents and enterprises is shown in Table 1.

### 3.2. Earnings Expectation Function Construction

Based on the above payment matrix, when a resident chooses the “cooperation” strategy, the expected benefits are

$${\mathrm{EA}}_{1}=y\left({\beta \varepsilon }^{\frac{1}{\lambda }}R+S-{\alpha \varepsilon }^{\frac{1}{\lambda }}C\right)+\left(1-y\right)\left(P-{\alpha \varepsilon }^{\frac{1}{\lambda }}C\right)$$

When residents choose the “non-cooperation” strategy, their expected return is

$${\mathrm{EA}}_{2}=x\left(-P\right)$$

When residents choose a mixed strategy, i.e., residents choose the “cooperation” and “non-cooperation” strategies, the average expected return is

$${\mathrm{EA}}_{3}={\mathrm{xEA}}_{1}+\left(1-x\right){\mathrm{EA}}_{2}=\mathrm{xy}\left({\beta \varepsilon }^{\frac{1}{\lambda }}R+S-{\alpha \varepsilon }^{\frac{1}{\lambda }}C\right)+x\left(1-y\right)+\left(1-x\right)y\left(-P\right)$$

Similarly, when an enterprise chooses the “cooperation” strategy, its expected benefits are

$${\mathrm{EB}}_{1}=x\left[\left(1-\beta \right)\varepsilon^{\frac{1}{\lambda }}R+S-\left(1-\alpha \right)\varepsilon^{\frac{1}{\lambda }}C\right]+\left(1-x\right)\left[P-\left(1-\alpha \right)\varepsilon^{\frac{1}{\lambda }}C\right]$$

When an enterprise chooses the “no cooperation” strategy, its expected benefit is

$${\mathrm{EB}}_{2}=x\left(-P\right)$$

When an enterprise chooses a mixed strategy, it is equal to an enterprise choosing both “cooperation” and “non-cooperation” strategies, and the average expected return is

$${\mathrm{EB}}_{3}={\mathrm{yEB}}_{1}+\left(1-y\right){\mathrm{EB}}_{2}=\mathrm{xy}\left[\left(1-\beta \right)\varepsilon^{\frac{1}{\lambda }}R+S-\left(1-\alpha \right)\varepsilon^{\frac{1}{\lambda }}C\right]+y\left(1-x\right)\left[P-\left(1-\alpha \right)\varepsilon^{\frac{1}{\lambda }}C\right]$$

### 3.3. Replicated Dynamic Equation Solving

Based on the above-expected return model, the dynamic replication equation for resident (A)’s choice of “cooperation” strategy can be derived as follows:

$$\begin{array}{cc} f\left(x\right) & =x\left({\mathrm{EA}}_{1}-{\mathrm{EA}}_{3}\right)=x\left(1-x\right)\left({\mathrm{EA}}_{1}-{\mathrm{EA}}_{2}\right)\\ & =x\left(1-x\right)\left[y\left({\beta \varepsilon }^{\frac{1}{\lambda }}R+S\right)-{\alpha \varepsilon }^{\frac{1}{\lambda }}C+P\right] \end{array}$$

The dynamic replication equation for the enterprise (B) to choose the “cooperation” strategy:

$$=y\left(1-y\right)\left[x\left(\left(1-\beta \right)\varepsilon^{\frac{1}{\lambda }}R+S\right)-\left(1-\alpha \right)\varepsilon^{\frac{1}{\lambda }}C+P\right]$$

From f(x) = 0, g(y) = 0, we can find the five local equilibrium points:

$$(0,0),\;(0,1),\;(1,0),\;(1,1),\left(\frac{\left(1-\alpha \right)\varepsilon^{\frac{1}{\lambda }}C-P}{\left(1-\beta \right)\varepsilon^{\frac{1}{\lambda }}R+S},\frac{{\alpha \varepsilon }^{\frac{1}{\lambda }}C-P}{{\beta \varepsilon }^{\frac{1}{\lambda }}R+S}\right)$$

The dynamic replication equation for resident and enterprise leads to the following:

(1) $$\frac{\mathrm{df}\left(x\right)}{x}=\left(1-2x\right)\left(y\left({\beta \varepsilon }^{\frac{1}{\lambda }}R+S\right)-{\alpha \varepsilon }^{\frac{1}{\lambda }}C+P\right)$$

(2) $$\frac{\mathrm{df}\left(x\right)}{y}=x\left(1-x\right)\left({\beta \varepsilon }^{\frac{1}{\lambda }}R+S\right)$$

(3) $$\frac{\mathrm{dg}\left(y\right)}{x}=y\left(1-y\right)\left(\left(1-\beta \right)\varepsilon^{\frac{1}{\lambda }}R+S\right)$$

(4) $$\frac{\mathrm{dg}\left(y\right)}{y}=\left(1-2y\right)\left(x\left(\left(1-\beta \right)\varepsilon^{\frac{1}{\lambda }}R+S\right)-\left(1-\alpha \right)\varepsilon^{\frac{1}{\lambda }}C+P\right)$$

The Jacobian matrix can be obtained by Equations (1)–(4):

$$J_{e}=\left[\begin{array}{cc} \left(1-2x\right)\left(y\left({\beta \varepsilon }^{\frac{1}{\lambda }}R+S\right)-{\alpha \varepsilon }^{\frac{1}{\lambda }}C+P\right) & x\left(1-x\right)\left({\beta \varepsilon }^{\frac{1}{\lambda }}R+S\right)\\ y\left(1-y\right)\left(\left(1-\beta \right)\varepsilon^{\frac{1}{\lambda }}R+S\right) & \left(1-2y\right)\left(x\left(\left(1-\beta \right)\varepsilon^{\frac{1}{\lambda }}R+S\right)-\left(1-\alpha \right)\varepsilon^{\frac{1}{\lambda }}C+P\right) \end{array}\right]$$

The value of the matrix determinant is:

$$\left|J_{e}\right|=\left(1-2x\right)\left(y\left({\beta \varepsilon }^{\frac{1}{\lambda }}R+S\right)-{\alpha \varepsilon }^{\frac{1}{\lambda }}C+P\right)\left(1-2y\right)\left(x\left(\left(1-\beta \right)\varepsilon^{\frac{1}{\lambda }}R+S\right)-\left(1-\alpha \right)\varepsilon^{\frac{1}{\lambda }}C+P\right)\;-x\left(1-x\right)y\left({\beta \varepsilon }^{\frac{1}{\lambda }}R+S\right)y\left(1-y\right)\left(\left(1-\beta \right)\varepsilon^{\frac{1}{\lambda }}R+S\right)$$

The traces of the matrix determinant are:

$${\mathrm{trJ}}_{e}=\left(1-2x\right)\left(y\left({\beta \varepsilon }^{\frac{1}{\lambda }}R+S\right)-{\alpha \varepsilon }^{\frac{1}{\lambda }}C+P\right)+\left(1-2y\right)\left(x\left({\beta \varepsilon }^{\frac{1}{\lambda }}R+S\right)-\left(1-\alpha \right)\varepsilon^{\frac{1}{\lambda }}C+P\right)$$

## 4. Model Discussion

According to the above evolutionary game model of residents and enterprises, the stability of residents and enterprises’ waste separation cooperation can be discussed in two cases.

### 4.1. Residents or Businesses Amortized Costs Are Less than the Respective Payment of Penalties for Breach of Contract

When the cost amortized by the resident or business is less than the respective penalty paid for default, that is an end.

According to the local stability analysis proposed by Friedman (1991) [39], four local equilibria in the system S = {(x,y); 0 ≤ x, y ≤ 1} are obtained, which are (0,0), (0,1), (1,0), and (1,1). The equilibrium results of the Jacobian Matrix are shown in Table 2.

**Figure 1:**
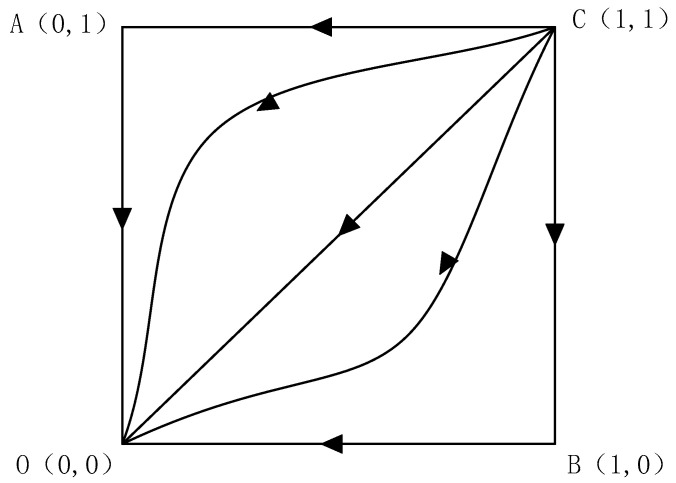
Phase diagram of the evolutionary game in which the amortized cost of residents or enterprises is less than the respective payment of default penalties.

From Table 2, it can be learned that when the respective amortized costs are smaller than the respective payment of default penalties, points (0,0), (0,1), and (1,0) are all unstable points, and point (1,1) is a partially stable point, as the residents or enterprises amortized costs are smaller than the respective payment of default penalties, the outcome of the evolutionary game between the two parties must be “cooperation”. This is also in line with the actual situation. The evolutionary phase diagram is shown in Figure 1.

### 4.2. Residents or Businesses Amortized Costs Are Higher than the Respective Payment of Penalties for Breach of Contract

When the cost amortized by the resident or business is greater than the respective penalty payment for breach of contract, that is $\left(1-\alpha \right)\varepsilon^{\frac{1}{\lambda }}C>P$ and ${\alpha \varepsilon }^{\frac{1}{\lambda }}C>P$.

It can be known that the system S = {(x,y); 0 ≤ x, y ≤ 1} has five local equilibria as (0,0), (0,1), (1,0), (1,1), and $\left(\frac{\left(1-\alpha \right)\varepsilon^{\frac{1}{\lambda }}C-P}{\left(1-\beta \right)\varepsilon^{\frac{1}{\lambda }}R+S},\frac{{\alpha \varepsilon }^{\frac{1}{\lambda }}C-P}{{\beta \varepsilon }^{\frac{1}{\lambda }}R+S}\right)$. The Jacobian matrix equilibrium results are shown in Table 3.

**Figure 2:**
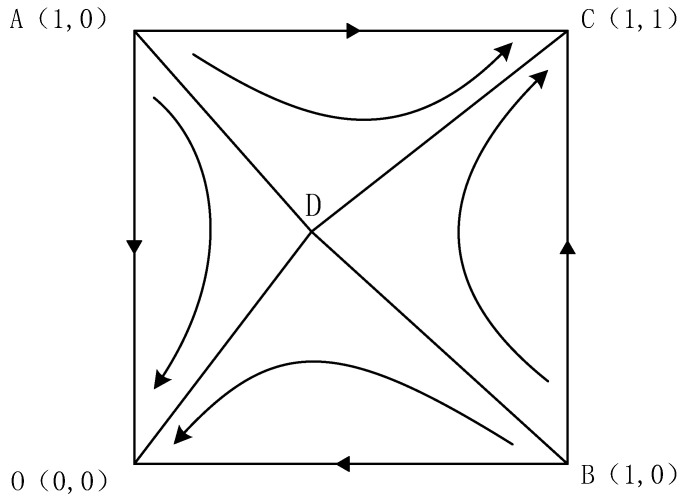
Evolution phase diagram when the amortized cost of residents or enterprises is greater than the default penalty paid by them, respectively.

From Table 3, we know that the points (0,0), (1,1) are local equilibrium points when the cost amortized by the resident or the enterprise is greater than the default penalty paid by each but correspond to different strategies, (cooperation, cooperation), (non-cooperation, non-cooperation), respectively. The points (0,1), (1,0) are local instability points, $\left(\frac{\left(1-\alpha \right)\varepsilon^{\frac{1}{\lambda }}C-P}{\left(1-\beta \right)\varepsilon^{\frac{1}{\lambda }}R+S},\frac{{\alpha \varepsilon }^{\frac{1}{\lambda }}C-P}{{\beta \varepsilon }^{\frac{1}{\lambda }}R+S}\right)$ is the saddle point, and the evolutionary phase diagram is shown in Figure 2.

As shown in Figure 2, when the initial value is located in the ACBD region, the system converges to C (1,1). The cooperative intention of residents and enterprises will evolve into the “cooperation” strategy. When the initial value is located in the ADBO region, the system converges to O (0,0). The cooperation intention of residents and enterprises will evolve into the “non-cooperation” strategy.

## 5. Numerical Analysis

### 5.1. Setting Parameters

Beijing Tianlong Tiantianjie Renewable Resources Recycling Co., Ltd. (Beijing, China) (after this, referred to as Tiantianjie) was established on 14 July 2007. It is the first pilot unit of Beijing Renewable resources recycling system construction and the leading company in Beijing Dongcheng District recycling system construction. At the beginning of 2016, the company selected 518 households in five residential buildings in Donghua City, Beijing, to carry out the waste separation and resource reduction work on a pilot basis. After nearly two years of pilot operation, the “two networks integration” mode of “Green Cat” waste separation and renewable resource recycling was finally determined. The “integration of two networks” mode is to curb the increment through the utilization of “dry waste” (recyclables) and reduce the stock through the utilization of “wet waste” (kitchen waste), to solve the problem of about 20% recyclables and about 30% kitchen waste in the residential waste structure. “Integration of the two networks” is mainly reflected in integrating human resources, information systems, logistics management, a publicity platform, and other aspects. After implementing the “integration of the two networks”, the amount of kitchen waste distributed in the streets of Donghua city has increased from about 40 tons per month at the beginning of 2018 to about 300 tons. The purity of distributed kitchen waste is more than 97%, and about 100 tons of renewable resources are recycled monthly. The residents’ waste separation participation rate has reached more than 80%. In 2020, this model covered eight streets, including Donghuashi Street, Longtan Street, Dongsi Street, Jinyu Road, and Tiantan Street, in the Dongcheng District of Beijing, serving nearly 100,000 households. The separation rate of kitchen waste in each street is more than 20%.

This paper visualizes the influence of the evolutionary game of residents’ and enterprises’ willingness to participate in waste separation cooperation. This paper uses Matlab2019a software to conduct simulation analysis, noting street residents as member A and separation and disposal enterprises as member B. By changing the values of different parameters, we observe and analyze the influence of each factor on residents’ and enterprises’ willingness to participate in waste separation.

The initial assignment of the parameters in the model is based on the reality of cooperation between residents and enterprises. Based on information from the financial statements of Tiantanjie company’s pilot 518-resident program. In this study, the coefficient of distributivity of the benefits of choosing the “cooperation” strategy is 0.5 for residents and 0.5 for enterprises. The cost of choosing the “cooperation” strategy is 0.3 for residents and 0.7 for enterprises; the complexity of the separation facilities in the street is 0.5, and the distributivity of the separation facilities is 0.6. The penalty paid by the “non-cooperation” party to the “cooperation” party due to breach of contract is 150,000; the government subsidy for both parties to choose the “cooperation” strategy is 80,000; the revenue after cooperation is 5 million. The expected cost is 3 million.

### 5.2. Sensitivity Analysis

#### 5.2.1. Impact of Changes in the Distributivity of Separation Facilities on Willingness to Cooperate

**Figure 3:**
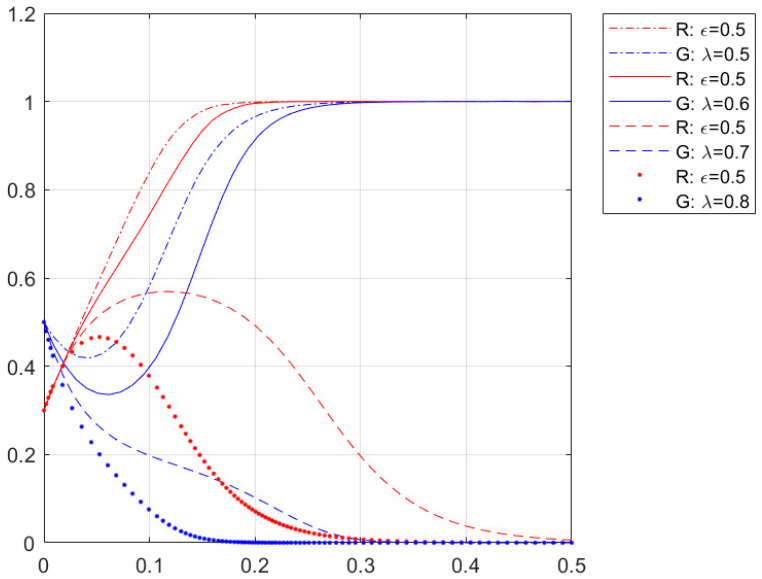
Impact on the willingness of residential enterprises to cooperate when the distributivity of separation facilities changes.

Figure 3 shows the simulation of changes in separation facilities’ distributivity on the residents’ enterprises’ willingness to cooperate, with other parameters constant. From Figure 3, the critical value of the distributivity of separation facilities λ is between 0.6 and 0.7. When the distributivity of separation facilities λ is less than this critical value, at this time, due to the low initial cooperative willingness of residents, the collective willingness of enterprises will have a slight decline. However, the residents and enterprises will eventually tend to cooperate and converge (1,1). At this time, the decrease of λ makes the cooperative willingness curve of residents and enterprises converge faster; when the distributivity of separation facilities λ is more significant than this critical value, at this time, the initial cooperative willingness of enterprises is higher. The collective willingness of residents is faster. When the distributivity of facilities λ is greater than the critical value, the initial willingness of enterprises to cooperate is higher, the willingness of residents to cooperate will rise slightly, and finally, the willingness of residents and enterprises to cooperate converges to (0,0), i.e., both residents and enterprises choose “non-cooperation”, at this time, the increase in the distributivity of separation facilities λ makes the cooperation curve of residents and enterprises converge to (0,0). This is since the cost paid by the enterprises’ side gradually increases as the distributivity of separation facilities increases in the game process. The simulation results show that when the complexity of separation facilities is certain, the lower the distributivity of separation facilities, the stronger the willingness to cooperate between residents and daily cleaning companies in Dongcheng District, Beijing streets.

#### 5.2.2. Impact of Changing Complexity of Separation Facilities on Willingness to Cooperate

**Figure 4:**
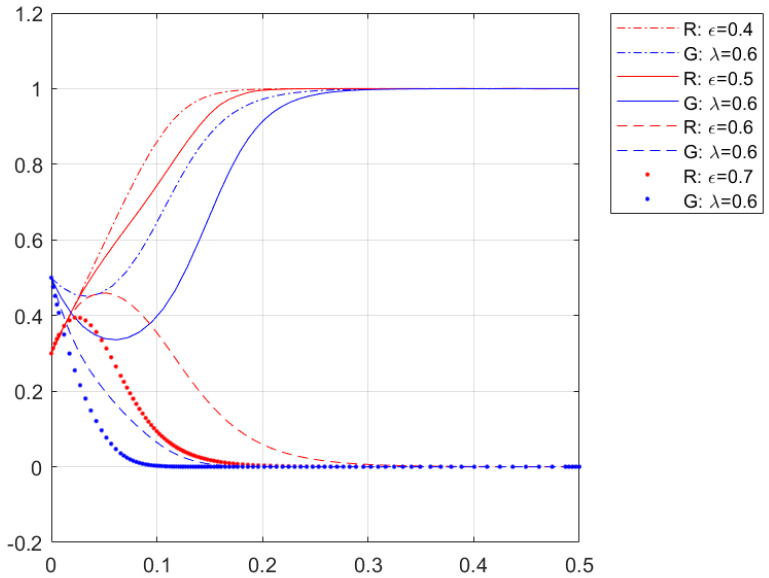
Impact on the willingness of residential enterprises to cooperate when the complexity of separation facilities changes.

Figure 4 shows the simulation of the change of separation facility complexity on residents’ and enterprises’ cooperation with other parameters held constant. From Figure 4, the critical value of separation facility complexity ε is between 0.5 and 0.6. When the separation facility complexity ε is less than this critical value, at this time, due to the low initial willingness of residents to cooperate, there will be a slight decline in the willingness of enterprises to cooperate, and finally the curve of the willingness of residents to cooperate converges to (1,1). At this time, the separation facility complexity ε decreases, which makes the curve of the willingness of residents to cooperate to converge faster. When categorical facility complexity ε is more significant than this critical value, due to the high initial cooperative willingness of enterprises, the cooperative willingness of residents will appear to rise briefly and then fall again, and finally the cooperative willingness curve of residents and enterprises converges to (0,0). When the categorical facility complexity ε rises, it will make the cooperative willingness curve of residents and enterprises converge to (0,0) faster, indicating that with the increase of categorical facility complexity, the cooperative willingness of residents and enterprises decreases. The reason is that when the complexity of separation facilities is low, the cost of cooperation paid by residents and enterprises is also low, although the benefit is less, but they have to pay a specific penalty for non-cooperation, so residents and enterprises will choose the “cooperation” strategy. When the complexity of separation facilities is high, the cost of cooperation paid by residents and enterprises increases, and residents and enterprises will choose the “non-cooperation” strategy. In the cooperation game, the greater the complexity of the separation facilities, the greater the cost of cooperation for the resident and the enterprises is, given the same distributivity of separation facilities. The simulation results show that the lower the complexity of the separation facilities, the more the residents of Beijing Dongcheng District are willing to cooperate with Tiantian Clean under certain distributivity of separation facilities.

#### 5.2.3. Impact of Simultaneous Changes in Distributivity and Complexity of Separation Facilities on Willingness to Cooperate

**Figure 5:**
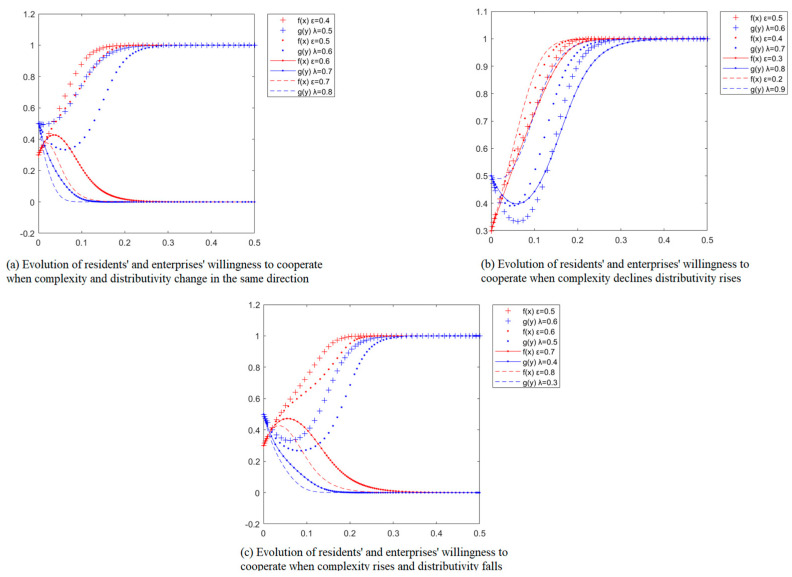
Impact of simultaneous changes in the distributivity and complexity of separation facilities on the willingness of residential enterprises to cooperate.

Figure 5a shows the simultaneous increase or decrease in the distributivity and complexity of the separation facilities of the waste separated by residential enterprises on the willingness of residential enterprises to cooperate, with other parameters held constant. When the distributivity λ and complexity ε are smaller than the critical values, the initial willingness of residents to cooperate decreases for a short period, and finally, the residents tend to cooperate. The equilibrium point converges to (1,1). When the distributivity λ and complexity ε are greater than the threshold value, the initial willingness to cooperate will be high, and the willingness to cooperate will rise for a short period. Moreover, finally, the residents and the enterprises choose the “non-cooperation” strategy, and the equilibrium point converges (1,1). The equilibrium point converges to (0,0). At this time, the increase of the distributivity of the separation facilities λ and complexity ε at the same time will lead to faster convergence of the cooperation curve of the resident and the enterprises to (0,0). This is because when the distributivity of facilities λ and complexity ε are not high, the cost of cooperation between residents and enterprises is low. The benefits are low, but the defaulting party will face the loss of penalty, so residents and enterprises will choose to cooperate; when the distributivity λ and complexity ε of separation facilities are high, the cost of cooperation between residents and enterprises increases, and the willingness of residents and enterprises to cooperate gradually weakens. In the separation game, the larger the distributivity of separation facilities λ, the higher the hardware cost, and the larger the complexity of separation facilities ε, the higher the cost of using and maintaining separation facilities. The simulation results show that when the distributivity of separation facilities λ and the complexity of separation facilities ε decrease at the same time, the willingness of residents and enterprises to cooperate becomes stronger; when the distributivity of separation facilities λ and the complexity of separation facilities ε increase at the same time, the willingness of residents and daily cleaning companies in Dongcheng District, Beijing to cooperate becomes weaker.

Figure 5b shows the simulation of the willingness of residents and enterprises to cooperate when the distributivity λ of separation facilities λ rises. The complexity ε decreases for separating waste separated by residents and enterprises, with other parameters constant. From Figure 5b, it can be obtained that when the distributivity λ of separation facilities rises and the complexity ε decreases, at this time, due to the low initial willingness of residents to cooperate, there will be a slight decline in the collective willingness of enterprises. Finally, the resident and the enterprises tend to cooperate. The equilibrium point converges to (1,1). At this time, the increase of λ and the decrease of ε make the cooperation curve of residents and enterprises converge to (1,1) faster. This is because the lower the complexity of the separation facilities, the lower the residential enterprises need to invest in the use and maintenance of the separation facilities.

In contrast, when the distributivity of the separation facilities is high, the enterprises have to pay a specific total hardware cost because the complexity of the separation facilities is low. The residents’ use cost is reduced, so the residential enterprises’ income is guaranteed. The simulation results show that when the complexity of separation facilities ε decreases and the distributivity λ increases, Beijing Dongcheng District streets residents are gradually more willing to cooperate with daily cleaning companies.

Figure 5c shows the simulation of decreasing separation facility distributivity λ and increasing complexity ε on residential enterprise cooperation in residential enterprise waste separation cooperation with other parameters. From Figure 5c, the critical values of simultaneous changes in the distributivity of separation facilities λ and complexity ε are 0.4–0.5 and 0.6–0.7, respectively. When the distributivity of separation facilities λ is more significant than this critical value and complexity ε is less than this critical value, at this time, due to the low initial willingness of residents to cooperate, the enterprises’ willingness to cooperate experiences a slight decline, and finally the residents and enterprises tend to choose the “cooperation” strategy, the equilibrium point converges to (1,1). At this time, the decline of separation facilities λ and the rise of complexity ε make the cooperation curve of residents and enterprises converge to (1,1) faster; when the distributivity of separation facilities λ is less than the critical value and complexity ε is greater than the critical value, at this time, because the initial willingness of enterprises to cooperate is high, the willingness of residents to cooperate experiences a slight increase. Finally, at this time, when the distributivity of facilities λ and the complexity ε are higher than the critical value, the residents’ willingness to cooperate tends to choose the “non-cooperation” strategy, and the cooperation curve of residents converges to (0,0). This indicates that as the distributivity of separation facilities λ decreases and complexity ε increases, the choice of residential enterprises gradually shifts from “cooperation” to “non-cooperation” as a result of when the distributivity of separation facilities is high but complexity is not high. When complexity is not high, the cost of using and maintaining residential enterprises is also low. When the distributivity of separation facilities is high, although the enterprises have to pay certain hardware costs, the complexity of separation facilities is not high. The use and maintenance costs are low, so the residents and the enterprises can gain a lot. However, when the distributivity of separation facilities is not high, and the complexity is high; the higher the complexity, the higher the cost of cooperation between residents and enterprises, and the more difficult it is to cooperate. According to the simulation, when the distributivity of separation facilities decreases and the complexity increases, the willingness of residents and daily cleaning companies to cooperate in Beijing’s Dongcheng District decreases.

## 6. Discussion

The convenience of waste separation facilities, as an actual vehicle and medium in waste separation, is of great practical significance to the impact of cooperative participation in waste separation. As a supplement to the past research, this paper divides the convenience of separation facilities into two analytical dimensions: distributivity of separation facilities and complexity of separation facilities. It establishes the game payment matrix of cooperative waste separation for residential enterprises based on the characteristics of two separate facilities, finds the solution of the equilibrium point of cooperative waste separation through the method of evolutionary game, analyzes the model stability conditions, and focuses on the different distributivity and complexity of separation facilities according to the impact on the willingness to cooperate in waste separation under different variations. The following perspectives are obtained:

(1) When the complexity of separation facilities is certain, the higher the distributivity of separation facilities, the lower the willingness of residential enterprises to cooperate in waste separation. When the complexity of separation facilities is certain, the higher the distributivity of separation facilities is, the stronger the willingness of residential enterprises to cooperate in waste separation. However, when the distributivity of separation facilities is high, the hardware cost that residential enterprises need to invest in increases, taking into account the diminishing marginal benefit effect. Therefore, residential enterprises should implement the layout of waste separation facilities in a planned and step-by-step manner. It is unreasonable to rely excessively on hardware investment in separation facilities to realize the increase in revenue.

(2) When the distributivity of separation facilities is certain, the willingness of residential enterprises to cooperate in waste separation rises and then falls with the increase in the complexity of separation facilities. When the complexity of separation facilities is low, the cost of using and maintaining them is low, making it easier for residential enterprises to form cooperation. Thus, it can be concluded that choosing waste separation facilities with a lower use threshold for waste separation cooperation, i.e., a lower use threshold for separation facilities, is more conducive to achieving a win-win situation for residential enterprises.

(3) When the distributivity and complexity of separation facilities change in the same direction, the willingness of residents and enterprises to cooperate in waste separation increases with the increase of distributivity and complexity and then decreases. When the distributivity and complexity of separation facilities reach a specific threshold value, the cost of waste separation cooperation increases sharply, and the willingness of residents and enterprises to cooperate decreases. Therefore, for waste separation cooperation projects with higher distributivity and complexity of separation facilities, government intervention is needed, such as special funding for the separation of private medical waste during the COVID-19 epidemic, to increase the willingness of residents and enterprises to cooperate in waste separation.

(4) When the distributivity and complexity of separation facilities change in the opposite direction, the willingness of residents to cooperate in waste separation varies with the convenience of the two separation facilities. As the distributivity of separation facilities decreases and complexity increases, the willingness of residential enterprises to cooperate gradually decreases; as the distributivity of separation facilities increases and complexity decreases, the willingness of residential enterprises to cooperate gradually becomes more vital. Therefore, residents and enterprises should use the separation facilities with lower use thresholds when cooperating in waste separation and design the distributivity plan of separation facilities according to each region’s topographical characteristics and population density. In addition, the government should adopt different support methods according to the actual situation of the convenience factors of the two separation facilities. Specifically, when the separation facilities are less distributed and more complex, it should decide whether it is necessary to increase the financial support according to the actual situation to realize the increased willingness of both parties to cooperate; when the separation facilities are more distributed and less complex, the government should avoid direct financial support and instead create a good business environment.

In summary, the convenience of separation facilities is an essential factor influencing the cooperation between residents and enterprises in waste separation, and the cooperation between residents and enterprises in waste separation is the only way to achieve waste separation. Waste separation facilities are an essential physical carrier for waste separation and a critical influencing factor. In the future, separation enterprises should put separation facilities in the community with reasonable layouts, lower the threshold of using separation facilities, and try to consider different people.

## 7. Conclusions

This research combines the results of the above discussion. There are important insights and suggestions for building a cooperative mechanism between residents and enterprises in waste separation.

(1) Enhance residents’ awareness of waste separation. The key is the attitude of individual residents and their awareness of waste separation. The participation of individual residents in waste separation synergy also does not necessarily mean that the synergy effect is improved, but also the quality and effect of residents’ participation in waste separation synergy. Therefore, relevant management departments should establish the school and non-school education systems, improve the atmosphere of residents’ waste separation awareness, establish a full-coverage waste separation education model, and effectively improve the quality and effectiveness of residents’ participation in waste separation cooperation to achieve efficient operation of the cooperative mechanism between residents and enterprises in waste separation.

(2) Increase the construction of waste separation facilities. The convenience of waste separation facilities, as an essential carrier and medium in the cooperative waste separation between residents and enterprises, is one of the factors affecting the effectiveness of cooperative waste separation. The convenience of separation facilities is mainly composed of distribution and complexity. When designing the distribution of separation facilities, we should consider the surrounding environment, population density, and other factors, i.e., the cost of the number of separation facilities and the convenience of residents, and set the distribution in a reasonable range. At the same time, when designing the complexity of separation facilities, we should consider the differences in residents’ literacy and learning ability. In addition, modern waste separation facilities are developing in the direction of digitalization.

(3) Continue to strengthen waste separation research. In this paper, we consider the convenience of waste separation facilities as a factor influencing waste separation cooperation, but in reality, more than the convenience of separation facilities affects waste separation cooperation. Due to the time constraints of data collection, we did not have sufficient time to conduct an empirical study. Therefore, we intend to conduct an empirical study on the collaboration between residents and enterprises based on the theory of planned behavior, an empirical study on the development of the waste separation industry in the context of digital transformation, and an empirical study on the spatial and temporal evolution of public reaction to waste separation management policies in the context of the digital economy in order to compensate for this limitation in our subsequent research work.

## Figures and Tables

**Table 1:** Waste separation cooperative payment matrix.

Residents (A)		**Enterprise (B)**	
		**Cooperation (y)**	**Non-Cooperation (1 − y)**
	Cooperation (x)	${\beta \varepsilon }^{\frac{1}{\lambda }}R+S-{\alpha \varepsilon }^{\frac{1}{\lambda }}C$	$-{\alpha \varepsilon }^{\frac{1}{\lambda }}C+P$
		$\left(1-\beta \right)\varepsilon^{\frac{1}{\lambda }}R+S-\left(1-\alpha \right)\varepsilon^{\frac{1}{\lambda }}C$	−P
	Non-cooperation (1 − x)	−P	0
		$-\left(1-\alpha \right)\varepsilon^{\frac{1}{\lambda }}C+P$	0

**Table 2:** Equilibrium results for residents or businesses with amortized costs less than the respective payment of default penalties.

Equilibrium Points	∣Je∣	trJe	Results
(0,0)	+	+	Saddle Point
(0,1)	+		Instability point
(1,0)	+		Instability point
(1,1)	+	-	ESS

**Table 3:** Residents or businesses amortizing costs are more excellent than the respective payment of penalties for breach of contract.

**Equilibrium Points**	**∣** **Je** **∣**	**trJe**	**Results**
(0,0)	+	−	ESS
(0,1)	+	+	Instability point
(1,0)	+	+	Instability point
(1,1)	+	−	ESS
$\left(\frac{\left(1-\alpha \right)\varepsilon^{\frac{1}{\lambda }}C-P}{\left(1-\beta \right)\varepsilon^{\frac{1}{\lambda }}R+S},\frac{{\alpha \varepsilon }^{\frac{1}{\lambda }}C-P}{{\beta \varepsilon }^{\frac{1}{\lambda }}R+S}\right)$	+	0	Saddle Point

## Data Availability

The data used to support the findings of this study will be available from the corresponding authors upon request.
